# A Transdisciplinary Approach to *Brucella* in Muskoxen of the Western Canadian Arctic 1989–2016

**DOI:** 10.1007/s10393-019-01433-3

**Published:** 2019-08-14

**Authors:** Matilde Tomaselli, Brett Elkin, Susan Kutz, N. Jane Harms, H. Ingebjørg Nymo, Tracy Davison, Lisa-Marie Leclerc, Marsha Branigan, Mathieu Dumond, Morten Tryland, Sylvia Checkley

**Affiliations:** 1 Faculty of Veterinary Medicine, University of Calgary, Calgary, AB Canada; 2 Canadian High Arctic Research Station, Polar Knowledge Canada, Cambridge Bay, NU Canada; 3 Department of Environment and Natural Resources, Government of Northwest Territories, Yellowknife, Inuvik, NT Canada; 4 Canadian Wildlife Health Cooperative, University of Calgary, Calgary, AB Canada; 5 Department of Environment, Animal Health Unit, Yukon Government, Whitehorse, YT Canada; 6 Research Food Safety and Animal Health, The Norwegian Veterinary Institute, Tromsø, Norway; 7 Department of Environment, Government of Nunavut, Kugluktuk, NU Canada; 8 Department of Arctic and Marine Biology, Research Group for Arctic Infection Biology, UiT – The Arctic University of Norway, Tromsø, Norway; 9 Alberta Provincial Laboratory for Public Health, Calgary, AB Canada

**Keywords:** *Brucella suis* biovar 4, *Ovibos moschatus*, Serology, Wildlife surveillance, Archives, Local knowledge, Participatory epidemiology, Public health

## Abstract

*Brucella* serostatus was evaluated in 3189 muskoxen sampled between 1989 and 2016 from various locations of the Canadian Arctic archipelago and mainland, near the communities of Sachs Harbour and Ulukhaktok, Northwest Territories, and Cambridge Bay and Kugluktuk, Nunavut. *Brucella* antibodies were found only in muskoxen sampled around Cambridge Bay, both on southern Victoria Island and on the adjacent mainland (Kent Peninsula). Consistent with participatory epidemiology data documented from local harvesters describing increased *Brucella*-like syndromes (swollen joints and lameness) and a decreased proportion of juveniles, the apparent *Brucella* seroprevalence in the sampled muskoxen of the Cambridge Bay area increased from 0.9% (95% CI 0.3–2.1) in the period of 1989–2001 to 5.6% (95% CI 3.3–8.9) in 2010–2016. The zoonotic bacteria *Brucella suis* biovar 4 was also cultured from tissues of muskoxen sampled on Victoria Island near Ulukhaktok in 1996 (*n* = 1) and Cambridge Bay in 1998, 2014, and 2016 (*n* = 3). Overall, our data demonstrate that *B. suis* biovar 4 is found in muskoxen that are harvested for food and by guided hunts on Victoria Island and Kent Peninsula, adding an important public health dimension to this study. Robust participatory epidemiology data on muskox health and diseases greatly enhanced the interpretation of our Cambridge Bay data and, combined with the serological and microbiological data, provide compelling evidence that the prevalence of *B. suis* biovar 4 has increased in this area since the late 1990s. This study enhances the available knowledge on *Brucella* exposure and infection in muskoxen and provides an example of how scientific knowledge and local knowledge can work together to better understand disease status in wildlife.

## Introduction

*Brucella suis* biovar 4 is a Gram-negative coccobacillus that is the etiologic agent of rangiferine brucellosis, a disease that is endemic in many barren-ground caribou (*Rangifer tarandus groenlandicus*) and reindeer (*Rangifer tarandus tarandus*) populations around the Arctic (Rausch and Huntley 1978; Tessaro and Forbes 1986; Thorne 2001; Godfroid et al. 2013, 2014; Carlsson et al. 2019). Rangiferine brucellosis is an important zoonosis that can result in a severe and highly debilitating disease in humans (Godfroid 2002). For humans, exposure to *B. suis* biovar 4 occurs through direct contact with infected animals either during butchering or through the consumption of undercooked meat, viscera, and bone marrow, as well as unpasteurized milk (OIE 2016). Although the current prevalence of *B. suis* biovar 4 in people is unknown, historic data for Alaska and Canada’s Arctic highlight that rangiferine brucellosis has occurred among northern peoples who consumed caribou (Meyer 1966; Huntley et al. 1963; Chan et al. 1989; Tessaro and Forbes 1986; Forbes 1991; Ferguson 1997). Rangiferine brucellosis continues to be an important public health concern in the Arctic, a place where many people rely on harvesting of caribou, muskoxen (*Ovibos moschatus*) and other wildlife for subsistence (CINE 2005; Meakin and Kurvits 2009; Tomaselli et al. 2018a). A human case of *Brucella suis* infection was recently documented in Ulukhaktok (Turvey et al. 2017), community of northern Canada included in our study area.

In caribou and reindeer, *B. suis* biovar 4 can cause granulomatous lesions primarily in bones, joints, and reproductive organs, leading to reproductive failure and increased susceptibility to predation (Thorne 2001). Evidence of *B. suis* biovar 4 exposure has been found in numerous carnivore species which prey on caribou (Neiland 1975; Zarnke et al. 2006); and natural infection with *B. suis* biovar 4 has been sporadically described in moose (*Alces alces*) and muskoxen that are sympatric with caribou (Honour and Hickling 1993; Edmonds et al. 1999; Gates et al. 1984; Forbes 1991; Tomaselli et al. 2016). More recently, within the scope of a participatory epidemiology study on muskox health and diseases in the community of Cambridge Bay (Victoria Island, Nunavut, Canada), harvesters reported increasingly observing signs of lameness and swollen joints in muskoxen (Tomaselli et al. 2018b). Additional hunter observations for the same time period included the decline of the local muskox population and a decreased proportion of juvenile muskoxen (Tomaselli et al. 2018b).

In response to these observations, as well as the detection of a subclinical case of *B. suis* biovar 4 in a hunter-killed muskox in 2014 (Tomaselli et al. 2016), we initiated a study to determine past and current exposure to, and occurrence of, *B. suis* biovar 4 in muskoxen in the western Canadian Arctic. This study is particularly relevant given that muskoxen are an important source of food and revenue for northern communities of Canada (Gunn and Adamczewski 2003; Tomaselli et al. 2018a; Cuyler et al. 2019).

We drew on a large sample size of archived sera and analyses from harvested and live-captured muskoxen from several locations of the western Canadian Arctic as well as ‘contemporary’ samples collected through our ongoing hunter-based muskox health sampling program in Cambridge Bay and Kugluktuk (Nunavut, Canada) (see Tomaselli 2018). The aims of this study were to (i) investigate over time and space the *Brucella* status of muskoxen using serology and opportunistic postmortem sampling, and (ii) to ‘triangulate’ this knowledge by combining and interpreting it with the available local knowledge on muskox health and diseases. This process of corroborating data using independent methods and knowledge sources is commonly used in participatory surveillance of livestock diseases as a way to enhance reliability of assessments (Mariner and Paskin 2000; Catley et al. 2012) and it can also be successfully applied to the veterinary surveillance of free-ranging wildlife populations (Tomaselli 2018).

This study increases the breadth and depth of knowledge on the occurrence of brucellosis in muskoxen, in part through the combined use of scientific knowledge and local knowledge. The transdisciplinary approach taken for this study is a practical example of the application of the ‘two-eyed seeing’ principle recently advocated for wildlife health (Kutz and Tomaselli 2019).

## Methods

### Brucella Serology

#### Blood Sample Collection

**Figure 1:**
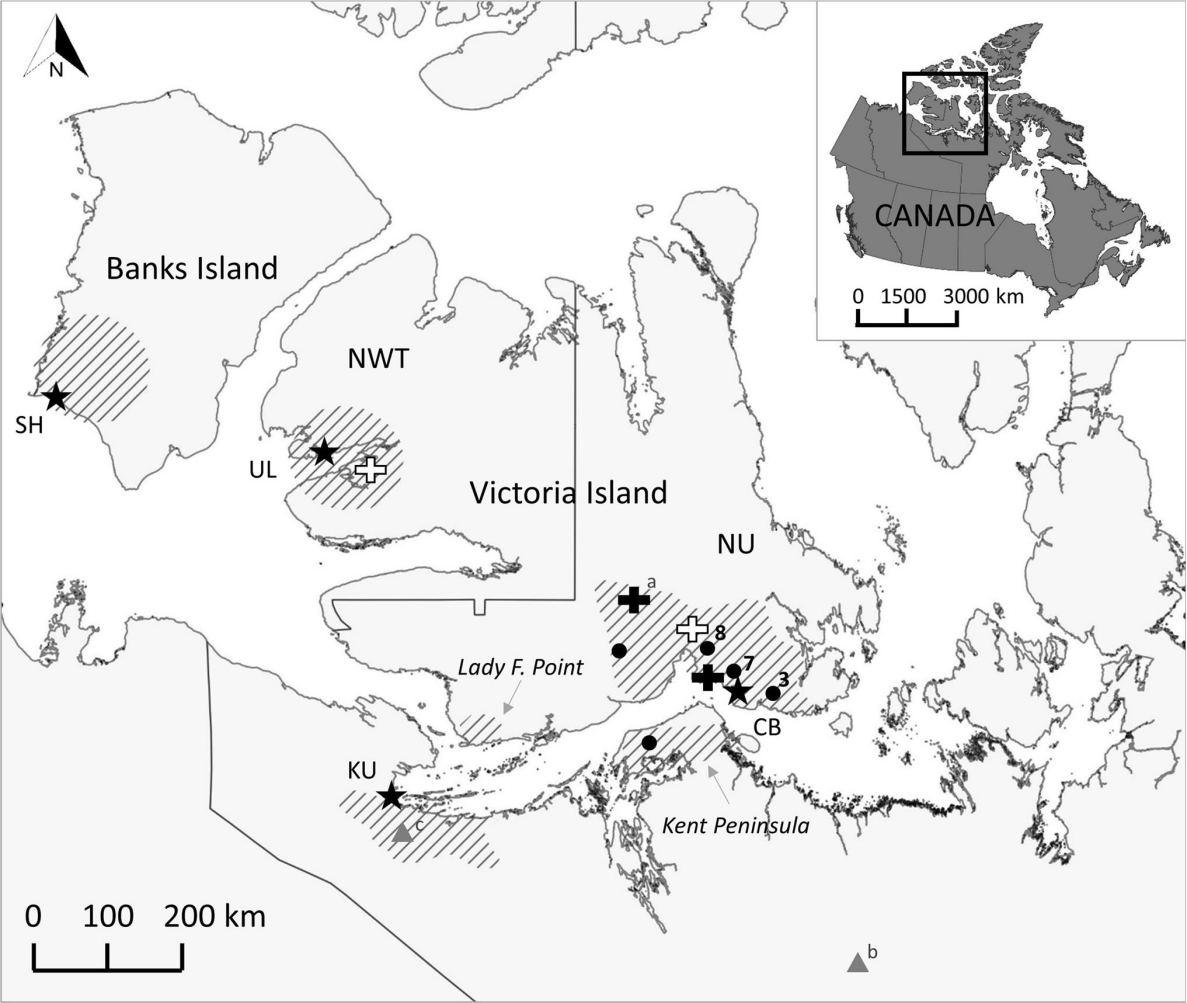
Area of study showing the locations where the samples were collected (line pattern fill) in proximity of the communities of Sachs Harbour (SH) and Ulukhaktok (UL), the Northwest Territories, and Cambridge Bay (CB) and Kugluktuk (KU), Nunavut (marked with a star). Locations of the *Brucella* positive muskoxen are marked with a black dot (only serology-positive blood samples), white cross (only microbiology positive tissue samples in which *Brucella suis* biovar 4 was isolated), and black cross (serology-positive blood samples coupled with microbiology positive tissue samples in which *B. suis* biovar 4 was isolated; with the letter a we refer to the microbiology result described in Tomaselli et al. 2016). When a georeferenced location represents more than one animal, a number indicates the sample size. For completeness, we finally indicate with gray triangles the locations of two male muskoxen from which *B. suis* biovar 4 was isolated in tissue samples and that are available in the published literature (b, Gates 1883; c, Forbes 1991).

Whole blood and/or blood-saturated filter paper (FP) strips were collected from muskoxen that were hunter-harvested (*n* = 3164), live-captured (*n* = 17), found dead (*n* = 7), and euthanized (*n* = 1) between 1989 and 2016 in various locations of the Canadian Arctic archipelago and mainland, near the communities of Sachs Harbour (SH, *n* = 1825) and Ulukhaktok (UL, *n* = 405), Northwest Territories, and Cambridge Bay (CB, *n* = 864) and Kugluktuk (KU, *n* = 95), Nunavut (Table 1; Fig. 1). During and prior to 2012, the vast majority of the samples were collected during commercial muskox harvests that occurred regularly on Banks and Victoria Islands near SH, CB, and UL, whereas near KU, samples were collected in conjunction with subsistence harvests and live-captures. After 2012, the majority of the samples were obtained through hunter-based sampling programs that were organized in CB and KU in association with outfitted hunts and subsistence harvests. A small number of additional samples were collected during opportunistic disease investigations near CB. Near KU, the collection of samples occurred on the mainland near the community, except for 24 and three muskoxen that were sampled on the southwest corner of Victoria Island (Lady Franklin Point) in 2010 and 2015, respectively. While the majority of the muskoxen sampled near CB were harvested on Victoria Island, 11 of the muskoxen sampled in 2016 were harvested on Kent Peninsula, on the adjacent mainland.

**Table 1:** Blood Samples of Muskoxen (*S* Serum, *FP* Filter Paper, *PL* Plasma) Included in the Study Presented by Location, Year(s) of Collection, and ‘Type’ of Muskoxen Sampled Divided in: a, Hunter-Harvested (^a^Commercial Harvest, ^b^Sport Hunts, ^c^Subsistence Harvest); b, Found Dead; c, Euthanized; d, Live-Captured.

Muskoxen sampled				Screening test			Confirmatory and new tests				Final status
Location	Year	Type	*N*	BPAT	STAT	*N* (+)	CFT	iELISA	cELISA	A/G iELISA	*N* (+)
Sachs Harbour	1999, 2000, 2003, 2006	a^a^	846	846^S^		0					0
Sachs Harbour	2008	a^a^	671	668^S^		6^S^		4^S^	6^S^	34^S,a^, 51^FP^ (48 paired)^b^	0
Sachs Harbour	2011	a^a^	243	243^S^		2^S^			2^S^		0
Sachs Harbour	2012	a^a^	65	62^S^		0				62^S,c^	0
Ulukhaktok	1994, 1996	a^a^	315	315^S^		0					0
Ulukhaktok	1999	a^a^	90	90^S^		0					0
Cambridge Bay	1989	a^a^	20	20^S^	20^S^	0					0
Cambridge Bay	1991	a^a^	20	20^S^		0					0
Cambridge Bay	1993, 1995, 2001	a^a^	246	246^S^		0					0
Cambridge Bay	1996	a^a^	130	130^S^		4^S^	4^S^				4^S^
Cambridge Bay	1998	a^a^	146	146^S^		1^S^	1^S^				1^S^
Cambridge Bay	2010	a^a^	55							55^P^	3^P^
Cambridge Bay	2011	a^a^	76							76^FP^	2^FP^
Cambridge Bay	2012	a^a^	42							14^S^, 28^FP^	1^FP^
Cambridge Bay	2014	a^b^	59							1^S^, 59^FP^ (1 paired)	1^S^, 4^FP^ (1 paired)
Cambridge Bay	2015	a^b^, a^c^,b	28,8,7							6^S^, 43^FP^ (6 paired)	1^S^, 5^FP^ (1 paired)
Cambridge Bay	2016	a^b^, a^c^,c	25,1,1							2^S^, 27^FP^ (2 paired)	1^S^, 2^FP^ (1 paired)
Kugluktuk	1991	a^c^,d	21,17	38^S^		0					0
Kugluktuk	2010	a^c^	24							24^FP^	0
Kugluktuk	2014	a^c^	16							16^FP^	0
Kugluktuk	2015	a^c^	13							13^FP^	0
Kugluktuk	2016	a^c^	4							4^FP^	0

The total number of animal sampled is indicated with *N*; while, the total number of samples and type of samples is specified under each test performed; finally, *N*(+) indicates the number of positive blood samples that were found after the screening tests (buffered plate agglutination test, BPAT; standard tube agglutination test, STAT) and the confirmatory tests (complement fixation test, CFT; indirect enzyme-linked immunoassay, iELISA; competitive enzyme-linked immunoassay, cELISA) and/or new test (protein A/G indirect enzyme-linked immunoassay, A/G iELISA) performed. Tested filter paper samples (FP) that had paired serum samples (S) are indicated in parenthesis.

^a^Sera tested also by BPAT at the time of sample collection.

^b^28 paired samples tested by A/G iELISA (FP eluates) and by BPAT (sera) at the time of sample collection, and 20 paired samples tested by A/G iELISA (both FP eluates and sera).

^c^59 sera tested also by BPAT at the time of sample collection

For the hunter-harvested muskoxen and the euthanized muskox, whole blood and/or blood-saturated FP strips were collected immediately after the animals were shot. Serum or plasma was obtained by collecting venous blood (jugular or femoral) into a Vacutainer^®^ tube with (i.e., ethylenediaminetetraacetic acid, EDTA) or without anticoagulant (for plasma and serum, respectively). In some cases, sterile falcon tubes were also used for the collection of whole blood. Filter paper samples were obtained by saturating the full length of Nobuto filter strips (Advantec MFS Inc., Dublin, California, USA) in venous blood as described by Curry et al. (2011). Immediately after collection, blood-saturated FP strips for each animal were placed into an antimicrobial-lined envelope (Quality Park, St. Paul, Minnesota, USA), except for the 19 FP strips collected in SH in 2008, which were stored in regular envelopes without antimicrobial-lining. For live-captured muskoxen, serum samples were obtained by collecting venous blood via jugular venipuncture into Vacutainer^®^ tubes after animals were chemically immobilized (Harms et al. 2012). Finally, for the seven muskoxen that were found dead near CB in 2015, blood samples (FP strips and/or Vacutainer^®^ tubes) were obtained from any site available for collection (i.e., heart, neck, leg). The times of death for these animals were estimated to be a few to several months prior to sample collection, and they had remained on the tundra under ambient temperatures below 0°C until sampled. Although they had remained ‘cool’, the carcasses were in varying states of decomposition and scavenging, thus the blood collection site depended on the state of carcass preservation.

After field collection, tube-collected blood was centrifuged for approximately 10 min at standard speed (i.e., G-Force and RPM as recommended by centrifuge manufacturer) and aliquots of serum or plasma were kept at − 20°C until tested, while blood-saturated FP samples were stored at − 20°C or air-dried overnight. All FPs were received at Faculty of Veterinary Medicine, University of Calgary (Alberta, Canada) and were stored at − 20°C or air-dried until testing (Curry et al. 2011, Curry et al. 2014a). Prior to testing, any frozen FP strips were also air-dried overnight at room temperature (Curry et al. 2011). One fully saturated FP strip for each dried sample was then re-suspended in phosphate-buffered solution following the protocol described by Curry et al. (2011) to obtain a FP elution estimated at 1:10 serum concentration. These were stored at − 20°C until antibody analysis.

#### ‘Historical’ Brucella Antibody Testing: BPAT and Confirmatory Ancillary Tests

A set of sera included in this study (CB 1989–2001; KU 1991; UL 1994–1999; SH 1999–2012) had been tested shortly after the respective times of collection for *Brucella* antibodies using the buffered plate agglutination test (BPAT) for screening as described by the Office International Des Epizooties (OIE 1996, 2016) (Table 1). Standard tube agglutination test (STAT) (Stemshorn 1985) was additionally used only for the CB collection of 1989. Sera that tested positive in the screening phase were further tested using ancillary tests: the complement fixation test (CFT) (Stemshorn 1985), or iELISA (Nielsen et al. 1994) and/or competitive enzyme-linked immunoassay (cELISA) (Nielsen et al. 1996) (Table 1). Sera were considered positive for *Brucella* antibodies if they remained positive after the ancillary-supplemental testing. Analyses were performed shortly after sample collection at the laboratories of the Canadian Food Inspection Agency (Brucellosis Centres of Expertise) in Lethbridge and Ottawa (Alberta and Ontario, Canada) except for sera collected near KU in 1991, and CB in 1989 and 1991. These were tested at the Health of Animals Laboratory in Saskatoon (Saskatchewan, Canada) following the same protocol.

#### ‘Contemporary’ Brucella Antibody Testing: A/G iELISA

The remaining sera, FP eluates and plasma samples (CB 2010–2016; KU 2010–2016) were tested for *Brucella* antibodies with a protein A/G indirect enzyme-linked immunoassay (A/G iELISA) (Nymo et al. 2013). Additionally, 93 BPAT-tested sera that were archived from the SH collection 2008 (*n* = 34) and 2012 (*n* = 59) were retested by A/G iELISA. Among the A/G iELISA-tested samples, there were 29 paired serum and FP samples (*n* = 20 SH 2008, *n* = 1 CB 2014, *n* = 6 CB 2015, *n* = 2 CB 2016). The paired serum and FP samples of the muskox harvested in CB in 2014 are from the case described in Tomaselli et al. (2016). The archived blood samples that were obtained from that case were here newly tested with the A/G iELISA. Finally, among the SH collection 2008, there were also 28 sera that were tested by BPAT at the time of collection and had paired FP samples that were newly tested by A/G iELISA (Table 1). Testing was performed blindly on both BPAT-tested sera and paired samples. The A/G iELISA testing was performed in 2017 at UiT—The Arctic University of Norway, Research Group for Arctic Infection Biology (Tromsø, Norway).

#### Serology Data Analyses and Interpretation

There is no information regarding the sensitivities and specificities of the tests used in this study for the detection of *Brucella* antibodies in muskoxen; therefore, cutoff values derived for reindeer and caribou (Gall et al. 2001; Nymo et al. 2013) were used. To assist with the interpretation of the A/G iELISA results, we report the percentage of positivity of the blood samples tested relative to the caribou bacteriology and serology-positive control (%*P* = [optical density sample/optical density positive control] × 100; Nymo et al. 2013). We compared the average %*P* values of the A/G iELISA negative and positive blood samples with the %*P* of the samples that were bacteriology positive (*n* = 2) and negative (*n* = 1). Based on tests results, we calculated the apparent prevalence (AP) together with the 95% confidence intervals computed using the Clopper–Pearson method (Brown et al. 2001).

Participatory epidemiology data on muskox demographics and morbidity (collectively defined as local knowledge) were available only for the Cambridge Bay area (see Tomaselli et al. 2018b) and were used to support analyses and interpretation of the CB samples. Cambridge Bay data obtained through this study were categorized into two time periods defined based on demography trends available through local knowledge, particularly a ‘pre-decline’ period from the 1990s to mid-2000s, and a ‘decline’ period from mid-2000s to the end of 2014 and onwards (Tomaselli et al. 2018b). The decline period was characterized by a major decrease in the number of muskoxen, and particularly the proportion of juveniles, as well as increasing observations of muskoxen with *Brucella*-like clinical signs such as swollen joints and lameness (Tomaselli et al. 2018b). The McNemar’s test for association of paired counts was used to test whether the *Brucella* seroprevalences of samples categorized in the ‘pre-decline’ and ‘decline’ periods were significantly different.

### Brucella-Cultured Muskoxen: Tissue Collection for Pathology and Microbiology Analyses

During the commercial harvests in UL in 1996, and CB in 1998, veterinarians inspected muskox carcasses and tissue samples with lesions that had *Brucella* infection listed as possible differential diagnosis were obtained from three and eight carcasses, respectively (Table 3). Shortly after the respective time of collection, tissue samples stored frozen (− 20°C) were submitted to the Canadian Wildlife Health Cooperative (CWHC) (University of Saskatchewan, Saskatoon, Canada) for further pathological and microbiological testing. Results of *Brucella* antibody testing (i.e., BPAT) on serum samples are also available for 10 of those 11 ‘historical’ cases.

In 2016, gross lesions consistent with *Brucella* infection were detected in two adult cows near CB (one sick cow that was euthanized by the Wildlife Officer and one that was harvested for subsistence). Shortly after collection, tissue samples stored frozen (− 20°C) were submitted for further testing to the CWHC (University of Calgary) and to the Canadian Food Inspection Agency Brucellosis National Reference Laboratory (Ottawa, Ontario, Canada). We also include here microbiology results of a previous case summarized in Tomaselli et al. (2016) that was hunted on Victoria Island (CB area) in 2014. Results of *Brucella* antibody testing (i.e., A/G iELISA) on paired serum and FP samples are also available for these ‘contemporary’ cases of 2014 and 2016.

## Results

### Brucella Serology

#### Overall Screening: ‘Historically’ and ‘Contemporary’ Tested Samples

Overall, the only blood samples that tested positive for *Brucella* antibodies were from hunter-harvested (*n* = 21) and euthanized (*n* = 1) muskoxen near the community of Cambridge Bay, both on Victoria Island and on Kent Peninsula, mainland (Table 1 and Fig. 1).

Among ‘historically’ tested samples (i.e., BPAT and confirmatory ancillary tests), *Brucella* antibodies were found by BPAT and confirmed by CFT in five CB sera collected on Victoria Island in 1996 and 1998 (Table 1). Of those, the CFT titers were 1/80 (*n* = 2), 1/160 (*n* = 1), and 1/640 (*n* = 1) for 1996 and 1/2560 (*n* = 1) for 1998. For SH samples, six and two sera collected in 2008 and 2011, respectively, were classified positive on BPAT but negative on the confirmatory tests performed (iELISA and/or cELISA) (Table 1). All other samples (SH, KU, UL) were negative for *Brucella* antibodies on BPAT (Table 1).

Among ‘contemporary’ tested samples (i.e., A/G iELISA), *Brucella* antibodies were found only in CB blood samples collected on Victoria Island between 2010 and 2016 (16/291) and on Kent Peninsula, mainland, in 2016 (1/11) (Table 1 and Fig. 1). All other samples (SH, KU) tested were classified negative for *Brucella* antibodies on A/G iELISA (Table 1).

#### Period Differences in Samples Tested from the Cambridge Bay Area

Serology data available for the CB area were further analyzed separated into the ‘pre-decline’ and ‘decline’ periods defined though participatory epidemiology. For muskoxen sampled in the period 1989–2001 (‘pre-decline’; BPAT-tested samples), the overall apparent *Brucella* seroprevalence was 0.9% (5/562, 95% CI 0.3–2.1), whereas, in the following sampling period 2010–2016 (‘decline’; A/G iELISA-tested samples), the overall apparent *Brucella* seroprevalence significantly increased to 5.6% (17/302, 95% CI 3.3–8.9; McNemar’s *χ*^2^ statistic 506, degrees of freedom 1, *p* value < 0.001).

#### Interpretation of Results from Serology Tests

To assist with the interpretation of the A/G iELISA results, we report in Table 2 the %*P* of the blood samples that were bacteriology positive (*n* = 2) and negative (*n* = 1). The %P of the rest of the negative and positive A/G iELISA-tested samples is also reported for comparison (Table 2). Regarding the comparison between A/G iELISA results of the 29 paired serum and FP samples (collected from the same individuals), there was complete agreement on their *Brucella* serostatus: 26 were seronegative and three were positive. Finally, regarding the comparison between diagnostic tests, the archived sera from SH 2008 (*n* = 34) and 2012 (*n* = 59) that were negative with the BPAT at the time of collection were also negative when retested with the A/G iELISA; and the 28 sera from SH 2008 that were negative with the BPAT at the time of collection had paired FP eluates that were also negative when tested with the A/G iELISA.

**Table 2:** Percentage of Positivity (%*P*) of Muskox Sera (S), Filter Paper Eluates (FP), and Plasma (PL) that were Classified Positive or Negative by A/G iELISA.

Sample identification	Status		*%P*		
	A/G iELISA	*B. suis* biovar 4	*S*	FP	PL
Hunted male, CB 2014	Positive	Positive^a^	37.43	40.40	n/a
Euthanized cow, CB 2016	Positive	Positive	41.58	25.68	n/a
Hunted cow, CB 2016	Negative	Negative	0.65	0.64	n/a
Remaining samples—positive	Positive	n/a	12.24 (*n* = 1)^b^	29.88 (SD 5.18; *n* = 12)	11.28 (SD 3.35; *n* = 3)
Remaining samples—negative	Negative	n/a	0.51 (SD 0.11; *n* = 115)	0.59 (SD 0.18; *n* = 326)	0.67 (SD 0.24; *n* = 52)

For each sample the %*P* was computed relative to the positive control used on the same plate (%*P* = [optical density sample/optical density positive control] × 100) where the positive control was from a microbiology and serology-positive caribou (Nymo et al. 2013). The first 3 samples were from animals confirmed by microbiology as either positive or negative for infection with *Brucella suis* biovar 4. For the remaining samples confirmatory microbiology was not available (n/a), thus are identified as positive or negative based on the A/G iELISA. For these samples the mean value is reported and the standard deviation (SD) and the number of samples tested (*n*) are specified in parenthesis.

^a^Tomaselli et al. (2016).

^b^Fund-dead cow, CB 2015 (%*P* in paired FP sample = 36.97).

### Brucella-Cultured Muskoxen

Of the 14 muskoxen that had postmortem lesions suspicious of brucellosis (including the case described in Tomaselli et al. 2016), four cultured positive for *B. suis* biovar 4 (Table 3). All culture-positive animals were from Victoria Island, one near UL in 1996 (a commercially-harvested muskox) and the remaining three near CB in 1998 (a commercially-harvested muskox), 2014 (a sport-hunted adult male; Tomaselli et al. 2016), and 2016 (a euthanized adult female) (Fig. 1). *Brucella* antibodies were not detected in blood samples of *Brucella* culture-negative muskoxen (Table 3). For the *Brucella* culture-positive muskoxen that had coupled serology results, *Brucella* antibodies were detected in paired sera and FP eluates that were tested with A/G iELISA (*n* = 2) but were not detected in the one serum sample that was tested with BPAT (CB 1998; Table 3).

**Table 3:** Microbiology and Serology Status of Samples (Tissues and Blood, Respectively) Collected from Muskoxen that had Gross Lesions with *Brucella* Infection Listed as a Possible Differential Diagnosis.

Location	Year	Identified lesion	Status	
			*B. suis* biovar 4	Serology
Ulukhaktok	1996	Lymphadenitis	Negative	Negative^1^
Ulukhaktok	1996	Nephritis, splenitis, lymphadenitis	Positive	n/a
Ulukhaktok	1996	Lymphadenitis	Negative	Negative^1^
Cambridge Bay	1998	Skin abscess	Negative	Negative^1^
Cambridge Bay	1998	Squamous cell carcinoma	Negative	Negative^1^
Cambridge Bay	1998	Lymphadenitis	Negative	Negative^1^
Cambridge Bay	1998	Lymphadenitis	Positive	Negative^1^
Cambridge Bay	1998	Fat abscess	Negative	Negative^1^
Cambridge Bay	1998	Lymphadenitis	Negative	Negative^1^
Cambridge Bay	1998	Lymphadenitis	Negative	Negative^1^
Cambridge Bay	1998	Lymphadenitis	Negative	Negative^1^
Cambridge Bay	2014	Metatarsal abscess	Positive^a^	Positive^2^
Cambridge Bay	2016	Bilateral abscesses in the vagina	Negative	Negative^2^
Cambridge Bay	2016	Granulomatous mastitis, endometritis, lymphadenitis, nephritis	Positive	Positive^2^

Bacteriology status of muskoxen was determined by culturing tissues with identified lesions; while serology status of muskoxen was determined by BPAT on sera (^1^), A/G iELISA on paired sera and filter papers eluates (^2^), or was not available (n/a).

^a^Tomaselli et al. (2016).

## Discussion

Our results, using contemporary and archived samples and data collected over almost 30 years, demonstrate that *B. suis* biovar 4 is increasingly found in muskoxen on Victoria Island and Kent Peninsula on the nearby mainland in the western Canadian Arctic. In addition, for the muskoxen of the Cambridge Bay area, serology data combined with available participatory epidemiology data (Tomaselli et al. 2018b) provide compelling evidence that the prevalence of *B. suis* biovar 4 has increased since the late 1990s. Although *Brucella* antibodies were not detected in the muskoxen sampled on Banks Island and the Kugluktuk area on the mainland, we cannot conclude that these locations are free of *B. suis* biovar 4 due to the limitations of study design (discussed later) and in absence of participatory epidemiology for triangulation. Our work confirms the importance of archived samples for understanding disease status and emergence in wildlife (Mörner et al. 2002; Hoberg et al. 2008; Ryser-Degiorgis 2013) and emphasizes the value of triangulating different data sources (i.e., scientific and local knowledge) to improve this understanding in the absence of perfect tests and study design (Tomaselli et al. 2018b), a common limitation when studying diseases in free-ranging animals (e.g., Wobeser 2007; Godfroid et al. 2010; Gilbert et al. 2013; Ryser-Degiorgis 2013). In a remote setting, such as the Arctic, this approach of acquiring data from multiple sources (active sampling, local knowledge, archival collections) can greatly strengthen future monitoring and surveillance efforts for rangiferine brucellosis and beyond (Tomaselli 2018).

### Considerations for the Interpretation of Brucella Serology Data

As typical for wildlife serological surveys, we encountered several challenges in the interpretation of serological data. Limitations to be considered are linked to the inability to use a probability sampling method, changing methodologies for *Brucella* serology screening and, most importantly, the lack of test validation. For example, although BPAT is one of the tests recommended by the OIE to screen for brucellosis in cattle with a sensitivity of 100% in this species (OIE 2016), the sensitivity of BPAT is unknown in muskoxen. When BPAT was validated to screen for brucellosis in other species, the sensitivities varied from 98% in reindeer (Gall et al. 2001) to 86% in bison (Nielsen and Gall 2001) and 77% in sheep (same subfamily muskoxen belong to; Nielsen and Gall 2001) leading to varying percentages of false negative results. Additionally, although the confirmatory tests used (i.e., CFT, iELISA, cELISA) all have a sensitivity of 100% for the detection of *Brucella* antibodies for caribou (Gall et al. 2001), they were used in series and not in parallel with BPAT; thus the overall sensitivity did not improve. We can’t exclude, therefore, that positive sera might have been missed in samples screened by BPAT; this possibility is reinforced considering that *Brucella* antibodies were not detected by BPAT in the serum of one muskox that was culture-positive for *B. suis* biovar 4 (CB 1998; Table 3).

With reference to the newly performed A/G iELISA, this test has been used extensively to screen for *Brucella* in muskoxen and other Arctic wildlife (Nymo et al. 2013, 2016). The A/G iELISA has been validated for *Brucella* antibody detection in blood of reindeer and caribou (sensitivity 100%, specificity 99.3%; Nymo et al. 2013) but not for muskoxen. In the present study, however, we observed a clear difference in the %*P* values (thus OD values) of the blood samples that were scored as negative or positive with A/G iELISA, which aligned with the %*P* of the blood samples of the culture-positive and culture-negative muskoxen (Table 2). Therefore, although the A/G iELISA has not been validated for muskoxen using a conventional methodology, the potential misclassification of the serostatus of the samples tested with A/G iELISA due to an inadequate cutoff value is unlikely in this study. As with other serological methods, A/G iELISA cannot distinguish between anti-*Brucella* antibodies and antibodies from cross-reacting bacteria that share common epitopes with *Brucella spp.* on the O-polysaccharide (Nymo et al. 2013). False positives from serological cross-reactions are a major problem for the interpretation of serological results from wildlife, including muskoxen, yet they are difficult to assess because little is known about co-infecting agents and their ability to cross-react in the different species (Nymo et al. 2013). Although false-positive results due to co-infections with cross-reacting agents cannot be excluded in this study, we note that A/G iELISA results matched the bacteriology results. The A/G ELISA *Brucella* results have also previously been shown to be in coherence with bacteriology results in caribou (Nymo et al. 2013).

One important challenge in this study was the use of different sample types (FP, serum, plasma), as well as different time of storage and methods of storage (i.e., filter papers only) prior testing. Contrary to BPAT-tested samples, which were tested shortly after time of collection, A/G iELISA-tested samples were stored for different periods of time prior to testing in 2017 (i.e., from a minimum of a few months to a maximum of 9 years). Additionally, some filter papers were stored frozen, while others were stored dried at room temperature. Curry et al. (2014a) evaluated in caribou and reindeer the effect of long-term storage of blood samples and storage methods of FPs on *Brucella* serology results and found comparable results to serum over a storage period of up to 2 years. Long-term stability studies on human serum samples archived at − 25°C have also shown that antibodies remain stable up to 25 years (Langseth et al. 2009); however, we cannot exclude that antibodies may deteriorate somewhat over time leading to false negatives. In this study, such a mechanism may have led to the underestimation of *Brucella* serostatus of samples collected in the Cambridge Bay and Kugluktuk areas from 2010 onwards. As a note we report that in 2014, shortly after sample collection, the serum of the muskox described in Tomaselli et al. (2016) was tested at the Brucellosis Center of Expertise, Ottawa and resulted highly positive for *Brucella* antibodies with the iELISA. In 2017, within the scope of this study, we tested the archived serum and paired filter paper (FP stored dried at room temperature since 2014) from that same animal with the A/G iELISA. Three years after field collection serum and FP were still highly positive for *Brucella* antibodies (see Table 3).

We were fortunate that paired FPs and sera were available for a subset of animals. The 100% results agreement obtained in paired FPs and sera indicates that FPs are valid samples for *Brucella* screening by A/G iELISA for muskoxen. These findings are consistent with what Curry et al. (2011) reported for caribou using iELISA. For the future, the easily implementable FP sampling can be an asset for increasing the field surveillance capacity for *Brucella* in harvested muskoxen. In this species, FPs might also be promising for ELISA screening for other pathogens as described for caribou (Curry et al. 2011; Curry et al. 2014a, b).

The change in testing approach over the sampling period 1989–2016 reflects the evolution in laboratory diagnostics for *Brucella* serology screening. We were able to compare the results from the A/G iELISA and BPAT testing for a subset of sera. The agreement between the two tests (100% in our subset of samples) makes us more confident in the results reported in this study despite the absence of test validation. Culture data were also invaluable for further interpretation of the serology testing. Animals that were culture positive for *B. suis* biovar 4 also were serologically positive by A/G iELISA. In contrast, one animal was negative on BPAT but positive by culture. In this case, the bacterium was isolated in a lymph node, but we cannot exclude that it was an early infection in which IgG were not yet produced. For future surveillance efforts, we suggest to prioritize the A/G iELISA for serology screening of *Brucella* in muskoxen. Combined implementation of serology with pathological and microbiological examinations is essential for the correct interpretation of disease status. Moreover, standardized sampling and analytical approaches are needed to evaluate changes in *Brucella* seroprevalence and/or disease spread over time and across localities.

#### Discussion of Results by Location

**Figure 2:**
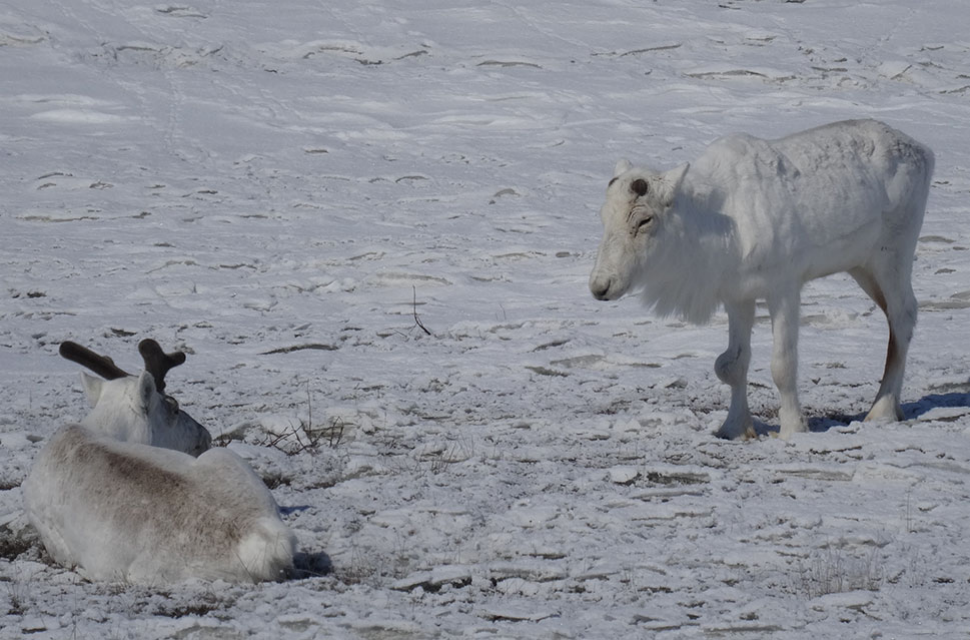
Two caribou of the Dolphin and Union herd photographed on May 08, 2019 on the mainland close to Kent peninsula and adjacent to Victoria Island. The caribou on the right has evident bilateral carpal hygromas—swollen joints—especially severe on the right leg: a typical sign of *Brucella suis* biovar 4 infection. This caribou, which also appears to have a severe subcutaneous infestation with warble fly larvae on its back, has a noticeable physiological delay for expected seasonal changes, such as growing antlers and shedding winter coat, compared to the caribou on the left, which appeared healthy. Photo: Inuit harvester, Candice Pedersen (Color figure online).

We only detected seropositive muskoxen in the Cambridge Bay area, both on Victoria Island and on Kent peninsula mainland. Our results suggest an increasing seroprevalence in this area; however, we are comparing BPAT-tested samples (1989–2001) with A/G iELISA-tested samples (2010–2016). To consider this increase valid based on our serology data alone, we have to assume that the tests have similar sensitivities and specificities and that the population tested is comparable in the two periods (i.e., same proportion of adults and juveniles, males and females). Based on available knowledge on test characteristics and sampling strategies, we cannot confirm these assumptions, thus limiting our confidence, based on serology data alone, that *Brucella* seroprevalence has truly increased. However, the triangulation of our serology data with historic and current participatory epidemiology and scientific data available for the same area provide supporting evidence that *B. suis* biovar 4 might truly be increasing in muskoxen on Victoria Island. Culture data from our study confirmed that *B. suis* biovar 4 is present in Victoria Island muskoxen. Additionally, the participatory epidemiology data gathered from Cambridge Bay harvesters align with what we would expect in a population where *B. suis* biovar 4 is circulating: a population decline with a decrease in the proportion of young animals suggesting reproductive failure, and typical *Brucella*-like syndromes such as swollen joints and limping animals (Tomaselli et al. 2018b). Furthermore, historic scientific information available for the Cambridge Bay area on muskoxen and sympatric caribou also supports that brucellosis may be increasing in this location. Blood samples from 120 muskoxen and 62 caribou of the Dolphin and Union herd collected between 1986 and 1990 on the southeastern Victoria Island were negative for *Brucella* antibodies (Gunn et al. 1991); however, a recent serological study of the Dolphin and Union caribou herd suggests that *Brucella* is now present in this species as well (Carlsson et al. 2019; Fig. 2). Whether the presence of *B. suis* biovar 4 in muskoxen on Victoria Island is associated with a spill-over event from the seasonally sympatric Dolphin and Union caribou herd, or if it has been circulating independently in muskoxen, cannot be determined based on our data. Finally, although the role of *Brucella* in the recent population declines remains uncertain, it has been implicated as influencing population dynamics elsewhere. For example, in the closely monitored caribou population of Southampton Island (Nunavut), the overall decline and decreased pregnancy rates were temporally associated with increasing *Brucella* seroprevalence (Campbell 2013). Additionally, increased *Brucella* seroprevalence was also found in a declining muskox population in Alaska (Afema et al. 2017).

Participatory epidemiology data documented through interviews of Cambridge Bay harvesters elucidated other mechanisms that could help further explain the observed change in demographics. These include the progressive deterioration of the body condition status of muskoxen and recent observation of *Orf*-like syndromes with orf virus confirmed through active sampling (Tomaselli et al. 2018b, 2016). The first mechanism can have a negative impact on pregnancy rates, while the second on calf survival. In the Cambridge Bay area, all these mechanisms—increased *Brucella* and orf virus exposure, and decline in body condition status—combined with widespread and unusual mortalities associated with *Erysipelothrix rhusiopathiae* (Kutz et al. 2015; Tomaselli et al. 2018b) and harvesting pressure may have interacted to produce the observed population decline. Further studies, including standardized sampling approaches and modeling, are required to understand the potential role of *Brucella* in the decline of the muskox population on Victoria Island.

In the Ulukhaktok area, although serology was negative on all historical samples tested, *B. suis* biovar 4 was isolated from one harvested muskox in 1996; contemporary samples were not available for this study. Given our results for the Cambridge Bay area, the detection of *Brucella* antibodies in sympatric caribou (Carlsson et al. 2019; Fig. 2) and the recent documentation of *Brucella suis* infection in one resident of Ulukhaktok (Turvey et al. 2017), it is possible that rangiferine brucellosis is circulating among the muskoxen of the Ulukhaktok area. We believe it is important to prioritize *Brucella* surveillance for the muskoxen of this area, including testing of contemporary samples and targeted participatory epidemiology studies.

We did not detect *Brucella* antibodies in the muskoxen sampled near Sachs Harbour (Banks Island) and Kugluktuk (mainland). However, given the limitations discussed for the interpretation of the serological data and the small sample size for the KU area, we cannot say definitely that brucellosis is absent from those areas. For Banks Island samples, although we cannot exclude that BPAT screening failed to detect *Brucella* antibodies, we are more confident in our results given a larger sample size and the fact serological testing was paired with veterinary postmortem inspections of carcasses which found no evidence of brucellosis (Elkin B., personal communication). On Banks Island, muskoxen do not have contact with barren-ground caribou (the most common hosts for the bacteria) but share their range with the Peary caribou (*Rangifer tarandus pearyi*) (Nagy et al. 1996). To date there are no reports of rangiferine brucellosis in Peary caribou on Banks Island (Species at Risk Committee 2012); however, there has been limited testing of this species for *Brucella* (Elkin B., personal communication) and local knowledge on brucellosis has not been documented. On the contrary, on the mainland, including the Kugluktuk area, available data already suggest that *B. suis* biovar 4 is present in muskoxen (Gates et al. 1984; Forbes 1991; Gunn et al. 1991; Fig. 1) and in sympatric barren-ground caribou (Gunn et al. 1991; Carlsson et al. 2019). For the future, documenting local knowledge from harvesters from Sachs Harbour and Kugluktuk will aid in better understanding historic and contemporary *Brucella* status of muskoxen in those areas. This is of great relevance especially for Banks Island given the continued and rapid decline of muskoxen (Kutz et al. 2017).

## Conclusion

*Brucella suis* biovar 4 is a pathogen with serious implication in the Arctic. Our study demonstrated that *B. suis* biovar 4 is present in muskoxen that are commonly harvested for food and through guided hunts on Victoria Island and the adjacent mainland. We initiated the study in response to local knowledge in the Cambridge Bay area that reported increased occurrence of clinical signs and demographic changes in muskoxen that were consistent with brucellosis. We were able to achieve a greater understanding of *Brucella* status in muskoxen in this area through the process of triangulation of data derived by active sampling, archived collections, and participatory epidemiology. The same level of understanding was not possible for the other locations where participatory epidemiology was not documented. This study reinforces that inference of disease status by relying on serology alone is challenging for wildlife and it provides evidence that a transdisciplinary approach that combines scientific and local knowledge can improve the understanding and strengthen the surveillance capacity for rangiferine brucellosis in the Arctic. Given the pathogenic potential of *B. suis* biovar 4 for both human and wildlife and the association of *Brucella* with population declines elsewhere, it is important to strengthen the surveillance for *B. suis* biovar 4 in muskoxen, understanding its epidemiology and impact, through the application of standardized sampling methods and testing combined with targeted participatory epidemiology studies. Collaborations with local harvesters and resource users not only will enhance the depth and breadth of understanding of *Brucella* status in local wildlife but also it will aid in implementing public health mitigation strategies that are locally customized.
